# Review of the Adverse Effects Associated with Dermal Filler Treatments: Part I Nodules, Granuloma, and Migration

**DOI:** 10.3390/diagnostics14151640

**Published:** 2024-07-30

**Authors:** Gi-Woong Hong, Hyewon Hu, Kathleen Chang, Youngjin Park, Kar Wai Alvin Lee, Lisa Kwin Wah Chan, Kyu-Ho Yi

**Affiliations:** 1 Samskin Plastic Surgery Clinic, Seoul 06577, Republic of Korea; 2 Division in Anatomy and Developmental Biology, Department of Oral Biology, Human Identification Research Institute, BK21 FOUR Project, Yonsei University College of Dentistry, 50-1 Yonsei-ro, Seodaemun-gu, Seoul 03722, Republic of Korea; wonhuh@yuhs.ac; 3 Harmony Aesthetic Clinic, Adelaide, SA 5000, Australia; harmonyaesthetica@gmail.com; 4 Obliv Clinic, Incheon 21998, Republic of Korea; youngjinp@gmail.com; 5 EverKeen Medical Centre, Hong Kong; alvin429@yahoo.com (K.W.A.L.); drchan.everkeen@gmail.com (L.K.W.C.); 6 Maylin Clinic (Apgujeong), Seoul 06001, Republic of Korea

**Keywords:** filler treatments, side effects, vascular complications, edema, granuloma

## Abstract

The increase in the use of filler treatments within minimally invasive cosmetic surgery has correspondingly escalated the variety and frequency of associated side effects. Initially, unregulated procedures led to primary side effects such as infections, foreign body reactions, and granuloma formation. However, severe vascular complications like skin and tissue necrosis and blindness have emerged as recognized risks. Side effects from filler treatments can range from mild to life-threatening, including edema, pain, tenderness, numbness, bleeding, bruising, hematoma, redness, erythema, pigmentation, allergic reactions, itching, pruritus, the Tyndall effect, asymmetry, irregularity, migration, skin and soft tissue infections, nodules, granulomas, and vascular compromise. These side effects are categorized into early and delayed types. Many complications, particularly those related to vascular abnormalities, are frequently linked to procedural issues, emphasizing the importance of understanding filler properties, injection techniques, and facial anatomy. Preventing side effects is ideal, but early detection and treatment are crucial. Recognizing potential side effects based on their timing and understanding appropriate preemptive treatment methods is essential. This discussion addresses non-vascular side effects, highlighting their onset, symptoms, and management strategies. The comprehensive understanding and careful management of these side effects are vital for minimizing complications and ensuring patient safety in filler treatments.

## 1. Introduction

The increase in the use of filler treatments within minimally invasive cosmetic surgery has correspondingly escalated the variety and frequency of associated side effects [1]. Prior to their widespread adoption, unregulated procedures often led to infections, foreign body reactions, and granuloma formation as primary side effects [2,3,4].

Side effects from filler treatments can vary from mild to life-threatening. Common side effects include edema (swelling), pain, tenderness, numbness, bleeding, bruising, hematoma, redness, erythema, pigmentation, allergic reactions, itching, pruritus, the Tyndall effect, asymmetry, irregularity, migration, skin and soft tissue infections, nodules, granulomas, and vascular compromise [2,3,5,6,7,8,9,10]. These side effects are categorized based on their onset into early and delayed types [8,11]. Many complications, particularly those related to vascular abnormalities, are frequently linked to procedural issues, underscoring the importance of a deep understanding of filler properties, injection techniques, and facial anatomy, particularly the vascular structures [2,3,7,12,13].

Recognizing potential side effects based on their timing and understanding appropriate preemptive treatment methods is essential [14,15,16,17,18,19,20,21,22,23,24]. Keywords including “filler injection”, “dermal filler”, “adverse effect”, “nodule”, “granuloma”, and “infection” were searched in the MEDLINE, PubMed, and Ovid databases for relevant studies published on clinical trials, diagnosis, and treatment. Some papers were further reviewed using a double-blinding approach, sample size, control usage, randomization usage, and objective endpoint measurements. All studies were classified according to the Oxford Center for evidence-based medicine evidence hierarchy [7]. The complications of the fillers were classified into early (hours and days) and mid and late complications (weeks to years post-procedure).

Summary of the Identified Literature:

In our review of the literature on the side effects and complications associated with dermal filler treatments, we identified a total of 123 relevant articles from various medical and cosmetic dermatology databases, including MEDLINE, PubMed, and Ovid. These studies encompassed a wide range of topics, including early and late-onset side effects, complications related to injection techniques, and patient-specific factors influencing outcomes. The identified literature provided detailed information on common side effects such as edema, bruising, hematoma, erythema, infections, nodules, granulomas, and vascular complications. Additionally, the literature offered insights into preventive measures and treatment protocols for managing these complications.

Number of Articles Excluded:

Out of the 123 initially identified articles, 74 were excluded from our review.

Reasons for Exclusion:

Lack of Relevance: Thirty articles were excluded because they did not directly address the side effects or complications of dermal filler treatments.

Insufficient Data: Twenty articles were excluded because of insufficient or incomplete data on side effects and complications.

Duplicate Studies: Fifteen articles were identified as duplicates and were excluded to avoid redundancy.

Poor Methodological Quality: Nine articles were excluded because of poor methodological quality, such as small sample sizes, lack of control groups, or non-randomized study designs.

By focusing on the remaining 49 high-quality articles, we ensured a comprehensive and reliable review of the side effects and complications associated with dermal filler treatments.

The aim of this study is to gather and comprehensively review the relevant literature on the side effects and complications associated with dermal filler treatments, with the goal of extracting valuable insights to better understand these adverse outcomes and improve clinical practices.

## 2. Early Side Effects

### 2.1. Post-Treatment Contour Irregularity

Contour irregularity represents the most common complication associated with the use of volumizing hyaluronic acid (HA) fillers. Depending on the underlying cause, these irregularities can range from transient lumpiness caused by immediate post-injection swelling, which typically resolves within a week, to uneven contours resulting from inadequate placement technique or insufficient injection volume. More persistent contour irregularities may develop weeks or months following an otherwise successful procedure because of individual physiological factors. Fortunately, such undesirable outcomes can often be corrected by the administration of hyaluronidase if an HA filler was used for the treatment.

Contour irregularity following an HA filler procedure generally falls into two categories as follows: those stemming from the inadequate placement of an HA filler and those developing gradually long after the initial procedure. Inadequate placement is often attributable to the practitioner’s lack of skill or the injection of insufficient volumes. Alternatively, irregularities can occur when only one localized area of the face is treated without considering the balance and harmony with the rest of the face. Cases where irregularities may not be apparent initially but appear over time are often due to factors such as subsequent aggregation of the HA filler caused by surrounding muscle contraction, gradual swelling of the HA filler due to its hydrophilicity, or variations in the longevity of HA fillers in different facial regions [5,25].

Overfilling certain facial areas can exacerbate contour irregularities. For instance, excessive filling of the lower pretarsal area during pretarsal roll augmentation can cause the injected volume to spill over into the adjacent infraorbital fat region, exacerbating puffiness under the eyes. Similarly, overcorrection of the canine fossa during nasolabial fold correction can cause excess volume to migrate upward, creating a doughy, pillowy appearance in the nasolabial fat pads. The periorbital area, with its thin skin, and the nasal and frontal regions, with limited space for HA filler placement, are particularly at risk for contour irregularities when suboptimal techniques are employed [15,26,27,28].

Irregularities in forehead contours can occur immediately after HA injection or develop with a time lag of 3–6 months post-treatment because of the contraction of surrounding muscles. Immediate unevenness is often the result of a suboptimal placement technique, such as failure to distribute the filler evenly across the forehead. Using a cannula instead of a needle can help create a smooth, even forehead contour, as its longer length allows for better reach into multiple areas across the forehead.

In the periorbital area, overcorrection of the tear trough can lead to the Tyndall effect or the appearance of disfiguring lumps. Unevenness can occur particularly when HA filler is added to the tear trough without addressing the volume deficit in the malar region below. Treatment of sunken eyes with fillers is also prone to developing contour irregularities, which may become more noticeable when the eyes are closed [29]. Therefore, moderate under-correction is advised when treating sunken eyes, aiming for only 60–70% correction with the eyes open. Pretarsal roll augmentation is also susceptible to lumpiness if the injection lacks smooth consistency or if too little volume is injected into loose, saggy lower pretarsal skin [30].

Occasionally, treatment may initially appear perfect because of post-injection edema, only to reveal subtle irregularities 1–2 weeks later as the swelling subsides. Patients should be informed of this possibility in advance to avoid misunderstandings and be advised that follow-up visits are scheduled to identify and correct any such irregularities. The primary purpose of volumizing fillers is to fill out areas of volume deficit or depletion, and achieving an aesthetically desirable outcome can be elusive when working with an insufficient amount of HA filler. Injecting too little filler can result in a loose and saggy appearance rather than a taut and tight one [5,25,31].

Key regions of the face where overcorrection can lead to undesirable outcomes include the following:

Nasal Dorsum: Overfilling can widen the nose, creating an unnatural appearance. Filler rhinoplasty involves injecting a fluid gel-type material to lift the overlying soft tissue. However, excess volume can spread sideways, broadening the nose [32].

Canine Fossa: Overcorrection during nasolabial fold treatment can result in an unnatural contour, blowing the malar cheeks out of proportion and giving a monkey-like appearance.

Forehead: Overfilling the central forehead without addressing the lateral forehead regions can lead to localized protrusion, while placing too much volume across the entire forehead can produce a disproportionately bulbous appearance [15,26,27,28].

Medial Malar and Zygomatic Region: Adding volume only in the medial malar to address infraorbital hollowness can cause lateral expansion of the cheeks, blending with the fullness already present in the lateral zygomatic region.

Pretarsal Roll: Overfilling can disrupt the balance and harmony of the eyes, creating a worm-like appearance if the roll becomes overly thick.

Lips: To prevent an overdone, duck-like appearance, minimal filler should be injected in a single session. In patients with a naturally protruded upper lip, conservative injection or avoiding the upper lip may be necessary [33]. The study by Taraneh et al. recommended using less than 1 cc of a filler to avoid issues [34].

The gradual appearance of irregularities over time is generally attributable to the subsequent clumping of the HA filler because of surrounding muscle activity or the hydrophilic nature of HA, which attracts water and gains volume. For instance, muscle contractions in regions of heavy facial animation, such as the forehead, glabella, and infraorbital regions, can cause the filler to shift and migrate from the implanted site, resulting in aesthetically displeasing bulges and irregularities. Protrusion of the hypertrophic medial muscular band of the orbicularis oculi muscle during smiling can accentuate the appearance of bulges, and repeated muscle contraction can cause the filler to clump up and protrude even when the face is at rest.

HA fillers’ hydrophilic nature can lead to post-injection swelling, particularly in areas with reduced dermal thickness such as the delicate periorbital region. Different types of HA fillers have different water absorption capacities, with monophasic HA fillers demonstrating a higher water retention capacity compared with biphasic HA fillers. This tendency to attract water can contribute to increased longevity but also poses a risk of undesired swelling and contour irregularities in delicate areas.

In conclusion, the management of post-treatment contour irregularities requires a thorough understanding of facial anatomy, proper injection techniques, and appropriate patient selection. Careful planning and execution, combined with the use of suitable fillers and adjunctive treatments, such as botulinum toxin injections, can help minimize the risk of contour irregularities and achieve optimal aesthetic outcomes.

### 2.2. Bruising and Hematoma

Bruising and hematoma are among the most prevalent early side effects of filler treatments, occurring immediately or within a few days of the procedure. Bruising is often visible immediately post-procedure if blood vessels are compromised during injection. Techniques like injecting in the supraperiosteal plane can mitigate but not completely prevent bruising [35].

If blood vessels around muscles or periosteum are deeply situated, bruising may not be immediately evident, complicating the application of adequate compression to prevent a hematoma. In such scenarios, hematomas may develop slowly and become apparent later [6,36,37,38,39]. Immediate application of sufficient compression is recommended even if minor bleeding is observed to prevent substantial bruising or hematoma later. Patients on anticoagulants or other medications that enhance bleeding should be advised to discontinue these medications several days prior to the procedure [4].

Factors influencing bruising and hematoma include needle size, treated layer, patient-specific factors (such as age, physical condition, vascular health, and existing conditions like liver disorders), and medications (e.g., aspirin, warfarin, NSAIDs, vitamin E, fish oil) [4]. To minimize bruising and hematoma, cold compresses before and after the procedure or mixing lidocaine with epinephrine for vasoconstriction during local anesthesia may be effective. Above all, a gentle and unhurried approach during all procedures is paramount [4,40].

When utilizing a cannula, creating space with partial tunneling before injection can increase the hematoma risk if tissue swelling indicates potential vascular damage. Immediate cannula removal and sufficient compression application are necessary in such cases. Compressing the area for at least 3–4 min to ensure no further swelling at the treatment site is recommended before either creating a new entry point for cannula insertion or postponing the procedure [4,40,41].

### 2.3. Edema

Properly administered filler injections can still result in transient edema, typically resolving within 2–3 days. Certain HA fillers may cause significant initial swelling because of their hyaluronic acid content and the properties of the filler, which promote water retention. Awareness of the degree of initial swelling for each filler type is crucial, and patients should be informed about potential swelling with specific fillers [37,42,43,44,45].

Distinguishing between hematoma from vascular damage and edema from water absorption by the filler is necessary. Hematomas are usually accompanied by bruising and are not uniform across the treatment area, often developing immediately post-procedure or within a few minutes. Excessive partial tunneling with a cannula can also cause severe swelling even without direct bleeding from vascular damage if soft tissue is excessively disrupted [41]. Minimizing partial tunneling and applying sufficient compression post-procedure can help prevent significant swelling.

Filler injections can also trigger a delayed hypersensitivity reaction days to months post-procedure, manifesting at the injection site or where the filler has migrated [8,9,20,45,46,47,48]. Symptoms of a delayed hypersensitivity reaction include severe swelling, erythema, and itching or pain at the injection site. Initial treatment typically involves administering steroids and antihistamines to alleviate symptoms. If symptoms persist or recur after initial improvement, removal of the filler may be necessary, as the reaction typically resolves once the filler is removed [5,11,36,49,50]. For HA fillers, the enzyme hyaluronidase can dissolve the filler; for non-HA fillers, manual expression through an incision or direct removal may be required [51,52,53,54]. If a nodule forms, injecting a diluted solution of triamcinolone or 5-FU into the nodule followed by vigorous massage can help break down the nodule before proceeding with removal techniques [54,55,56].

### 2.4. Infection

Symptoms such as a burning sensation, erythema, or swelling within 2–3 days post-filler injection suggest a potential infection at the injection site [57]. Preventing procedural-related infections requires thorough disinfection of the treatment area and the use of sterile medical gloves during the procedure [9,49,58,59]. Post-procedure antibiotics are sometimes prescribed as a preventative measure.

For treating infections, oral antibiotics are typically administered. Common choices include Amoxicillin + Clavulanate or Cephalexin; for penicillin-allergic patients, Ciprofloxacin is an alternative [5,20,52]. If an infection persists despite initial antibiotic treatment, the presence of an abscess should be evaluated. If an abscess is suspected, incision and drainage followed by pus culture to identify the appropriate antibiotic are necessary [49,54]. For an inflammatory nodule, antibiotics effective against such infections, such as Clarithromycin + Moxifloxacin, Ciprofloxacin, or Minocycline, are used [48].

Herpes infections around the mouth require differential diagnosis, especially in immunocompromised patients who might experience herpes virus recurrence after filler injections near the mouth. Symptoms include a tingling sensation at the injection site, followed by vesicle and crust formation [10,45,60]. Differentiating this from skin necrosis, which presents with a reticular pattern and color change in the skin, is crucial. Prophylactic antiviral treatment should be considered for patients with a history of frequent herpes recurrences at the time of the procedure. Prophylactic treatment typically involves prescribing Valacyclovir 500 mg twice daily for three days, while treatment for an actual viral infection involves prescribing 2000 mg of Valacyclovir twice a day for one day [46,61].

It is also necessary to distinguish from skin reactions occurring during a delayed hypersensitivity reaction, which may include itching and diffuse erythema but typically not a burning sensation [10,45,62].

### 2.5. Side Effects Related to Injection Depth and Amount

Selecting a layer rich in blood vessels for injection naturally leads to bruising, hematoma, and other symptoms such as pain, tenderness, numbness, and abnormal facial expressions due to damage to facial sensory and motor nerves. Excessive or shallow injections of filler can cause visible skin markings or a bluish appearance due to the Tyndall effect, as well as redness, erythema, or pigmentation [35,37,63].

Inappropriate injection techniques or excessive use can cause the filler to migrate, leading to asymmetry, lumpiness, lumps, beading, or nodules. Excessive molding or improper techniques after using highly elastic, firm fillers for total facial contouring can reduce the volumizing effect and cause deformities due to excessive molding. The direction of applied pressure can cause the filler to move, leading to indented or uneven areas. If the filler does not return to its original shape after counter-pressure, special care must be taken during molding [40,64].

## 3. Mid- to Late-Stage Side Effects

### 3.1. Erythema with Neovascularization

Mild erythema often occurs at the injection site immediately following the procedure and generally resolves within several days. However, there are instances where diffuse erythema, characterized by circular or oval patches, manifests days or weeks post-filler injection. This delayed diffuse erythema primarily occurs in the superficial layers close to the skin surface but may also develop at any depth where tissues are tightly adhered without intervening loose spaces [44]. Such erythema arises because of increased pressure from the surrounding firm tissues impacted by the filler, which can impede blood circulation and lead to compensatory neovascularization [37,44,58].

In severe cases, the management of erythema may involve applying steroid creams and employing vascular lasers for symptom relief. However, prolonged use of steroid creams should be approached with caution as they can dilate capillaries and potentially worsen the symptoms [35].

It is crucial to differentiate diffuse erythema from symptoms indicative of impending necrosis and soft tissue infection. Erythema associated with impending necrosis typically presents 1–3 days post-treatment, displaying a distinctive reticular pattern in shades of red or light purple, which requires immediate intervention for necrosis [65,66].

Skin and soft tissue infections following filler treatments generally appear 2–3 days post-procedure. The erythema in these infections is typically dark red in the center, fading in a gradient towards the edges, and is not symmetrical across the face. These infections often induce a burning sensation and swelling, necessitating antibiotic therapy [47,49].

### 3.2. Post-Inflammatory Hyperpigmentation (PIH)

PIH may occur at injection sites following inflammation triggered by fillers, particularly in individuals with Fitzpatrick skin types IV and VI. To mitigate this risk, it is recommended to minimize the number of entry points and utilize the smallest possible needles for cannula insertions.

If pigmentation develops, treatment options include skin-lightening agents such as hydroquinone or laser therapies using Q-switched Nd:YAG or Pico-second Nd:YAG lasers, aimed at reducing the visibility of hyperpigmentation (Table 1) [44,46].

### 3.3. Nodule and Granuloma

Filler injections may occasionally induce dermatitis-like symptoms due to allergic reactions to components such as collagen, lidocaine, and carboxymethylcellulose (CMC), found in products like Radiesse, Facestem, Elansé, and Rykoll [11,45]. Additionally, adverse reactions can occur if the pH of the filler significantly deviates from the body’s natural pH. In the case of hyaluronic acid (HA) fillers, unbound cross-linkers may act as toxins and elicit inflammatory responses [8,11].

While pure HA fillers are generally considered biocompatible and safe, as they are absorbed without triggering immunological tissue reactions, mixtures containing additional components can still provoke these immune responses. The advancement in filler technology has positioned them as a favorable alternative to fat grafting procedures. The development of firm HA fillers, used in substantial quantities to augment facial volume, can compress surrounding tissues, disrupt blood circulation, and potentially trigger infections or biofilm inflammatory responses (Table 2). Such prolonged and severe reactions may lead to immunological tissue or foreign body reactions, culminating in granuloma formation [53,67]. The overuse of firm fillers can heighten the frequency and severity of allergic reactions due to tissue compression.

Patients previously experiencing allergic reactions to any fillers, including HA fillers, are at an increased risk of eliciting immunological hypersensitivity reactions in subsequent treatments. Historically, allergic reactions to non-hyaluronic acid components in HA fillers were rare with minimal usage for wrinkle removal. However, the recent trend in employing large quantities of firm HA fillers to maximize facial volume has led to an uptick in non-antibody-dependent hypersensitivity reactions, akin to type IV reactions. It is speculated that excessive amounts of cross-linking agents like BDDE in HA fillers may separate from the HA molecules during injection and degradation, potentially leading to immunological side effects. This frequent use of BDDE-cross-linked HA fillers has prompted FDA reviews concerning the safety of HA fillers based on cumulative usage amounts [42,45,68].

Following the initial swelling from HA filler injections, the hygroscopic nature of HA fillers induces hydration. The degree of hydration varies with the product, yet excessive hydration can yield unpredictable outcomes. Fillers causing minimal and consistent hydration are deemed superior. If an HA filler causes more frequent and extensive swelling than typically expected, enzymatic dissolution is recommended to mitigate the formation of a thicker-than-normal capsule around the filler, which heightens the risk of bacterial infection [5,9,44] Subsequent factors like viral infections, bacterial conditions such as tonsillitis or sinusitis, skin inflammations, vaccinations, dental procedures, or immune-stimulating medications might act as immunological triggers, increasing the likelihood of delayed hypersensitivity reactions (Table 3) [11].

Granulomas resulting from foreign body reactions are categorized into early and late stages. Early-stage granulomas emerge from processes such as epidermal fibrosis leading to hypertrophic scars, irregular reactions, allergic responses causing telangiectasia, immune hypersensitivity reactions forming lumps or nodules, and inflammatory responses that result in abscesses (Table 4). Late-stage granulomas are typically classified into cystic, edematous, and sclerotic types, presenting as erythematous cysts, distinctly swollen areas, and bluish nodules, respectively [44,46,48,67].

## 4. Discussion

The Autoimmune/Inflammatory Syndrome Induced by Adjuvants (ASIA) syndrome, first defined in 2011, describes adverse immune reactions following exposure to adjuvants—substances that enhance immune response. ASIA can be triggered by bioimplants like hyaluronic acid (HA), leading to immune dysregulation. This syndrome involves mechanisms of both innate and adaptive immunity, causing dysregulation of T and B lymphocytes, inflammation, and tissue damage, eventually resulting in autoimmunity. Predisposing genetic factors, such as specific HLA antigens, play a significant role in the development of ASIA. Clinical manifestations may include muscle weakness, joint pain, fatigue, and other systemic symptoms, often developing years after exposure to the adjuvant. Recognizing and diagnosing ASIA can be challenging because of its nonspecific symptoms and the potential delay in onset [69,70].

Another common side effect is Facial Overfilled Syndrome (FOS), which is characterized by an excessive use of fillers, leading to a distorted and heavy appearance. Common manifestations include “flowerhorn” foreheads, “sunset” eyes, “chipmunk” cheeks, “witch” chins, and “pillow” faces. This syndrome results from attempts to combat anatomical aging with filler treatments, causing rapid volume changes and skin tightening. FOS arises because of excessive filler use, poor injection techniques, misunderstanding of the aging process, anatomical disparities, and issues with filler pressure and migration. Prevention methods involve tailored assessment and treatment plans that consider muscle activity, facial movements, and fat layer thickness using ultrasound imaging. It is crucial to consider anatomical factors to avoid overcorrection by respecting natural bone structures and tissue distribution, particularly in different ethnicities. Minimizing filler use is essential, and alternative approaches such as energy-based devices and polymer reinforcement of facial ligaments can be employed. Proper injection techniques that focus on myomodulation to adjust muscle activity and using appropriate products to withstand shear stress and compression are also important. Ongoing education and training ensure that physicians are well-versed in facial anatomy and the aging process to prevent and address FOS [26,27,28,71].

The increase in the use of filler treatments within minimally invasive cosmetic surgery has correspondingly escalated the variety and frequency of associated side effects. Initially, unregulated procedures often led to primary side effects such as infections, foreign body reactions, and granuloma formation. However, as these treatments have become mainstream, severe vascular complications such as skin and tissue necrosis and blindness have emerged as recognized risks.

Side effects from filler treatments can vary from mild to life-threatening. Common side effects include edema (swelling), pain, tenderness, numbness, bleeding, bruising, hematoma, redness, erythema, pigmentation, allergic reactions, itching, pruritus, the Tyndall effect, asymmetry, irregularity, migration, skin and soft tissue infections, nodules, granulomas, and vascular compromise. These side effects are categorized based on their onset into early and delayed types. Many complications, particularly those related to vascular abnormalities, are frequently linked to procedural issues, underscoring the importance of a deep understanding of filler properties, injection techniques, and facial anatomy, particularly the vascular structures.

Bruising and hematoma are among the most prevalent early side effects of filler treatments, occurring immediately or within a few days of the procedure. Bruising is often visible immediately post-procedure if blood vessels are compromised during injection. Techniques like injecting in the supraperiosteal plane can mitigate but not completely prevent bruising. Immediate application of sufficient compression is recommended even if minor bleeding is observed to prevent substantial bruising or hematoma later. Patients on anticoagulants or other medications that enhance bleeding should be advised to discontinue these medications several days prior to the procedure. Factors influencing bruising and hematoma include needle size, treated layer, patient-specific factors (such as age, physical condition, vascular health, and existing conditions like liver disorders), and medications. To minimize bruising and hematoma, cold compresses before and after the procedure or mixing lidocaine with epinephrine for vasoconstriction during local anesthesia may be effective.

Properly administered filler injections can still result in transient edema, typically resolving within 2–3 days. Certain HA fillers may cause significant initial swelling because of their hyaluronic acid content and the properties of the filler, which promote water retention. Awareness of the degree of initial swelling for each filler type is crucial, and patients should be informed about potential swelling with specific fillers. Distinguishing between hematoma from vascular damage and edema from water absorption by the filler is necessary. Hematomas are usually accompanied by bruising and are not uniform across the treatment area. Minimizing partial tunneling and applying sufficient compression post-procedure can help prevent significant swelling.

Symptoms such as a burning sensation, erythema, or swelling within 2–3 days post-filler injection suggest a potential infection at the injection site. Preventing procedural-related infections requires thorough disinfection of the treatment area and the use of sterile medical gloves during the procedure. Post-procedure antibiotics are sometimes prescribed as a preventative measure. For treating infections, oral antibiotics are typically administered. If an infection persists despite initial antibiotic treatment, the presence of an abscess should be evaluated. For an inflammatory nodule, antibiotics effective against such infections, such as Clarithromycin + Moxifloxacin, Ciprofloxacin, or Minocycline, are used. Herpes infections around the mouth require differential diagnosis, especially in immunocompromised patients. Prophylactic antiviral treatment should be considered for patients with a history of frequent herpes recurrences at the time of the procedure.

Mild erythema often occurs at the injection site immediately following the procedure and generally resolves within several days. However, delayed diffuse erythema, characterized by circular or oval patches, may manifest days or weeks post-filler injection. This type of erythema arises because of increased pressure from the surrounding firm tissues impacted by the filler, which can impede blood circulation and lead to compensatory neovascularization. Management may involve applying steroid creams and employing vascular lasers for symptom relief.

Post-inflammatory hyperpigmentation (PIH) may occur at injection sites following inflammation triggered by fillers, particularly in individuals with Fitzpatrick skin types IV and VI. Minimizing the number of entry points and utilizing the smallest possible needles for cannula insertions can reduce this risk. If pigmentation develops, treatment options include skin-lightening agents such as hydroquinone or laser therapies using Q-switched Nd or Pico-second Nd lasers.

Filler injections may occasionally induce dermatitis-like symptoms due to allergic reactions to components such as collagen, lidocaine, and carboxymethylcellulose (CMC). Adverse reactions can occur if the pH of the filler significantly deviates from the body’s natural pH. Granulomas resulting from foreign body reactions are categorized into early and late stages. Early-stage granulomas emerge from processes such as epidermal fibrosis leading to hypertrophic scars, irregular reactions, allergic responses causing telangiectasia, immune hypersensitivity reactions forming lumps or nodules, and inflammatory responses that result in abscesses. Late-stage granulomas are typically classified into cystic, edematous, and sclerotic types.

Filler treatments, while effective for cosmetic enhancement, carry risks that require thorough understanding and careful management. The categorization of side effects into early and delayed types helps in timely identification and intervention. With the increasing prevalence of filler treatments, ongoing education on injection techniques, filler properties, and anatomical knowledge is crucial for minimizing complications and ensuring patient safety. The next part of this discussion will delve deeper into vascular complications associated with filler treatments, providing a comprehensive overview of their prevention and management.

Our review offers a comprehensive overview of dermal filler side effects and complications, utilizing a wide range of studies from reputable databases. Categorizing side effects into early and delayed types aids in timely identification and intervention. We employed the Oxford Center for Evidence-based Medicine evidence hierarchy to ensure high-quality evidence and provided practical insights into preventive measures and treatment protocols.

However, limitations include the exclusion of non-English studies, potential heterogeneity among the included studies, a focus on short-term outcomes with limited long-term data, and possible publication bias favoring positive outcomes.

Future research should focus on long-term studies to better understand the persistent effects of dermal fillers. Developing standardized reporting protocols for side effects and complications will improve research quality. Further investigation into the biological mechanisms behind adverse effects, including immune responses and filler migration, is necessary. Studies should include diverse populations to assess the impact of variables such as ethnicity, age, and gender. Comparative effectiveness research can identify the safest and most effective fillers and techniques. Advanced imaging techniques like MRI and ultrasound can provide detailed insights into post-injection changes. Including patient-reported outcomes will help gauge the impact on quality of life and aesthetic satisfaction. Addressing these areas will enhance the safety, efficacy, and patient satisfaction of dermal filler treatments.

## 5. Conclusions

In conclusion, our comprehensive review highlights the wide range of side effects and complications associated with dermal filler treatments, emphasizing the importance of timely identification and intervention. While our study provides valuable insights and practical recommendations for clinicians, it also underscores the need for further research, particularly long-term studies, standardized reporting protocols, and a deeper understanding of the biological mechanisms involved. Future studies should consider diverse populations and employ advanced imaging techniques to enhance the safety and efficacy of dermal fillers, ultimately improving patient satisfaction and outcomes in cosmetic dermatology.

## Figures and Tables

**Table 1:** Potential adverse events and complications according to onset time.

Early onset (Hours to 7 Days)	Mid to Late Onset (1 Weeks to Years)
Bruising and hematoma	Improper placement (migration or displacement)
Pain, tenderness, and numbness	Neovascularization
Swelling (initial swelling and hydration) and late edema	PIH (post-inflammatory hyperpigmentation)
Redness, erythema, and pigmentation	Chronic infection with biofilm inflammation
Allergic reaction (itching and pruritus)	Nodule with immunological hypersensitivity reaction
Skin marking (superficial injection)	Granuloma with foreign body reaction
Tyndall effect (bluish discoloration) by superficial injection of large volume	
Asymmetry, irregularities, visible lumps, or bumps and beading	
Infection	
Vascular compromise (skin and tissue necrosis, blindness, etc.)	

**Table 2:** Conditions that aggravate biofilm infection after soft tissue filler injection.

Conditions Aggravating Biofilm Infection
1. Allergy and normal foreign body reaction.
2. Improper disinfection of skin.
3. Pathogen existing normally on skin.
4. Decreased immunity and bad injection technique.
5. Type of filler including shape and size of filler particle, hydrophobicity, degree of purification.
6. Depth and region of injection. The probability of biofilm infection is increased with deeper injections.
7. Diameter of needle.
8. Duration of filler.

**Table 3:** Immunological triggers that may cause hypersensitivity after filler injection.

Immunological Triggers That May Cause Hypersensitivity after Filler Injection
1. Viral and bacterial illness (streptococcal infection, flu-like syndrome, herpes zoster infection, sinusitis or tonsillitis, dermatitis such as acne, etc.).
2. Various vaccination (influenza, COVID-19 vaccine, etc.).
3. Dental treatment or scaling for periodontitis.
4. Drugs (Interferon, etc.).
5. Overall tired body condition (cold, overnight, etc.).

**Table 4:** Early and late foreign body granuloma.

Early Foreign Body Granuloma	Late Foreign Body Granuloma
1. Hypertrophic scar and irregularity by superficial fibrotic process.	1. Cystic type (erythematous).
2. Telangiectasia by allergic reaction.	2. Edematous type (swelling).
3. Lump and nodule formation by immunological hypersensitivity reaction.	3. Sclerosing type (bluish confined nodule).
4. Inflammatory reaction with lump or allergies -> long-lasting redness -> sterile abscesses.	

## Data Availability

The data are available request to the corresponding author.

## References

[1] Woodward J., Khan T., Martin J. (2015). Facial Filler Complications. Facial Plast. Surg. Clin. N. Am..

[2] Nettar K., Maas C. (2012). Facial filler and neurotoxin complications. Facial Plast. Surg..

[3] Park K.H., Kim Y.K., Woo S.J., Kang S.W., Lee W.K., Choi K.S., Kwak H.W., Yoon I.H., Huh K., Kim J.W. (2014). Iatrogenic occlusion of the ophthalmic artery after cosmetic facial filler injections: A national survey by the Korean Retina Society. JAMA Ophthalmol..

[4] Lemperle G., Rullan P.P., Gauthier-Hazan N. (2006). Avoiding and treating dermal filler complications. Plast. Reconstr. Surg..

[5] Ozturk C.N., Li Y., Tung R., Parker L., Piliang M.P., Zins J.E. (2013). Complications following injection of soft-tissue fillers. Aesthet. Surg. J..

[6] Beleznay K., Carruthers J.D., Humphrey S., Jones D. (2015). Avoiding and Treating Blindness From Fillers: A Review of the World Literature. Dermatol. Surg..

[7] Hong J.H., Ahn S.J., Woo S.J., Jung C., Chang J.Y., Chung J.H., Han M.K. (2014). Central retinal artery occlusion with concomitant ipsilateral cerebral infarction after cosmetic facial injections. J. Neurol. Sci..

[8] Artzi O., Loizides C., Verner I., Landau M. (2016). Resistant and Recurrent Late Reaction to Hyaluronic Acid-Based Gel. Dermatol. Surg..

[9] Alhede M., Er Ö., Eickhardt S., Kragh K., Alhede M., Christensen L.D., Poulsen S.S., Givskov M., Christensen L.H., Høiby N. (2014). Bacterial biofilm formation and treatment in soft tissue fillers. Pathog. Dis..

[10] Seok J., Hong J.Y., Park K.Y., Kim B.J., Seo S.J., Kim M.N., Hong C.K. (2016). Delayed immunologic complications due to injectable fillers by unlicensed practitioners: Our experiences and a review of the literature. Dermatol. Ther..

[11] Beleznay K., Carruthers J.D., Carruthers A., Mummert M.E., Humphrey S. (2015). Delayed-onset nodules secondary to a smooth cohesive 20 mg/mL hyaluronic acid filler: Cause and management. Dermatol. Surg..

[12] (2024). Hong G-W, Hu H, Chang K, Park Y, Lee KWA, Chan LKW, Yi K-H. Adverse Effects Associated with Dermal Filler Treatments: Part II Vascular Complication. Diagnostics.

[13] Park S.W., Woo S.J., Park K.H., Huh J.W., Jung C., Kwon O.K. (2012). Iatrogenic retinal artery occlusion caused by cosmetic facial filler injections. Am. J. Ophthalmol..

[14] Yi K.H., Lee J.J., Hur H.W., Bae H.K., Kim H.-J. (2022). Hyaluronic acid filler injection for deep nasolabial folds: A novel intraoral approach. Clin. Anat..

[15] Lim T.S., Wanitphakdeedecha R., Yi K.H. (2024). Exploring facial overfilled syndrome from the perspective of anatomy and the mismatched delivery of fillers. J. Cosmet. Dermatol..

[16] Yi K.H., Oh W., Kim H.M., Ahn H.S., Hu H., Kim H.J. (2023). Anatomical proposal for hyaluronic acid filler injection in subzygomatic arch depression: A dual-plane injection technique. Clin. Anat..

[17] Schelke L.W., Decates T.S., Velthuis P.J. (2018). Ultrasound to improve the safety of hyaluronic acid filler treatments. J. Cosmet. Dermatol..

[18] Lemperle G., Duffy D.M. (2006). Treatment options for dermal filler complications. Aesthet. Surg. J..

[19] Choi D.-Y., Bae J.-H., Youn K.-H., Kim W., Suwanchinda A., Tanvaa T., Kim H.-J. (2018). Topography of the dorsal nasal artery and its clinical implications for augmentation of the dorsum of the nose. J. Cosmet. Dermatol..

[20] Rohrich R.J., Monheit G., Nguyen A.T., Brown S.A., Fagien S. (2010). Soft-tissue filler complications: The important role of biofilms. Plast. Reconstr. Surg..

[21] Bray D., Hopkins C., Roberts D.N. (2010). A review of dermal fillers in facial plastic surgery. Curr. Opin. Otolaryngol. Head. Neck Surg..

[22] Rohrich R.J., Bartlett E.L., Dayan E. (2019). Practical Approach and Safety of Hyaluronic Acid Fillers. Plast. Reconstr. Surg. Glob. Open.

[23] Kim S.N., Byun D.S., Park J.H., Han S.W., Baik J.S., Kim J.Y., Park J.H. (2014). Panophthalmoplegia and vision loss after cosmetic nasal dorsum injection. J. Clin. Neurosci..

[24] Kang S.H., Moon S.H., Kim H.S. (2020). Nonsurgical Rhinoplasty With Polydioxanone Threads and Fillers. Dermatol. Surg..

[25] Trinh L.N., Grond S.E., Gupta A. (2022). Dermal Fillers for Tear Trough Rejuvenation: A Systematic Review. Facial Plast. Surg..

[26] Cotofana S., Gotkin R.H., Frank K., Lachman N., Schenck T.L. (2020). Anatomy Behind the Facial Overfilled Syndrome: The Transverse Facial Septum. Dermatol. Surg..

[27] Lim T. (2024). Facial Overfilled Syndrome. Dermatol. Clin..

[28] Schelke L., Harris S., Cartier H., Alfertshofer M., Doestzada M., Cotofana S., Velthuis P.J. (2023). Treating facial overfilled syndrome with impaired facial expression-Presenting clinical experience with ultrasound imaging. J. Cosmet. Dermatol..

[29] Huang Y.L., Liang B.C. (2024). Evolving Forehead Augmentation: A Five-step Approach with High G Prime Hyaluronic Acid. Plast. Reconstr. Surg. Glob. Open.

[30] Wang D., Huang X., Zhou Y., Gong M., Lu Y., Ni M. (2023). Cosmetic augmentation of lower-lid pretarsal roll with injection of autologous fat for Asians. J. Plast. Reconstr. Aesthetic Surg..

[31] Park Y.J., Cha J.H., Han S.E. (2021). Maximizing Thread Usage for Facial Rejuvenation: A Preliminary Patient Study. Aesthetic Plast. Surg..

[32] Park S.Y., Kim S.B., Suwanchinda A., Yi K.H. (2024). Non-surgical rhinoplasty through minimal invasive nose thread procedures: Adverse effects and prevention methods. Ski. Res. Technol..

[33] Keramidas E., Rodopoulou S., Gavala M.I. (2021). A Safe and Effective Lip Augmentation Method: The Step-by-Step Φ (Phi) Technique. Plast. Reconstr. Surg. Glob. Open.

[34] Yazdanparast T., Samadi A., Hasanzadeh H., Nasrollahi S.A., Firooz A., Kashani M.N. (2017). Assessment of the Efficacy and Safety of Hyaluronic Acid Gel Injection in the Restoration of Fullness of the Upper Lips. J. Cutan. Aesthet. Surg..

[35] Ablon G. (2016). Understanding How to Prevent and Treat Adverse Events of Fillers and Neuromodulators. Plast. Reconstr. Surg. Glob. Open.

[36] Kim S.B., Hu H., Bae H., Yi K.H. (2024). Anatomy of the temporal region to guide filler injections. Surg. Radiol. Anat..

[37] DeLorenzi C. (2013). Complications of injectable fillers, part I. Aesthet. Surg. J..

[38] DeLorenzi C. (2014). Complications of injectable fillers, part 2: Vascular complications. Aesthet. Surg. J..

[39] Snozzi P., van Loghem J.A.J. (2018). Complication Management following Rejuvenation Procedures with Hyaluronic Acid Fillers-an Algorithm-based Approach. Plast. Reconstr. Surg. Glob. Open.

[40] Signorini M., Liew S., Sundaram H., De Boulle K.L., Goodman G.J., Monheit G., Wu Y., Trindade de Almeida A.R., Swift A., Vieira Braz A. (2016). Global Aesthetics Consensus: Avoidance and Management of Complications from Hyaluronic Acid Fillers-Evidence- and Opinion-Based Review and Consensus Recommendations. Plast. Reconstr. Surg..

[41] Tansatit T., Apinuntrum P., Phetudom T. (2017). A Dark Side of the Cannula Injections: How Arterial Wall Perforations and Emboli Occur. Aesthetic Plast. Surg..

[42] Broder K.W., Cohen S.R. (2006). An overview of permanent and semipermanent fillers. Plast. Reconstr. Surg..

[43] Ono S., Ogawa R., Hyakusoku H. (2010). Complications after polyacrylamide hydrogel injection for soft-tissue augmentation. Plast. Reconstr. Surg..

[44] Narins R.S., Coleman W.P., Glogau R.G. (2009). Recommendations and treatment options for nodules and other filler complications. Dermatol. Surg..

[45] Alijotas-Reig J., Garcia-Gimenez V. (2008). Delayed immune-mediated adverse effects related to hyaluronic acid and acrylic hydrogel dermal fillers: Clinical findings, long-term follow-up and review of the literature. J. Eur. Acad. Dermatol. Venereol..

[46] Curi M.M., Cardoso C.L., Curra C., Koga D., Benini M.B. (2015). Late-onset adverse reactions related to hyaluronic Acid dermal filler for aesthetic soft tissue augmentation. J. Craniofac Surg..

[47] Goldan O., Garbov-Nardini G., Regev E., Orenstein A., Winkler E. (2008). Late-onset infections and granuloma formation after facial polylactic acid (New-Fill) injections in women who are heavy smokers. Plast. Reconstr. Surg..

[48] Ledon J.A., Savas J.A., Yang S., Franca K., Camacho I., Nouri K. (2013). Inflammatory nodules following soft tissue filler use: A review of causative agents, pathology and treatment options. Am. J. Clin. Dermatol..

[49] Hanke C.W., Higley H.R., Jolivette D.M., Swanson N.A., Stegman S.J. (1991). Abscess formation and local necrosis after treatment with Zyderm or Zyplast collagen implant. J. Am. Acad. Dermatol..

[50] Park T.H., Seo S.W., Kim J.K., Chang C.H. (2012). Clinical experience with polymethylmethacrylate microsphere filler complications. Aesthetic Plast. Surg..

[51] Lee S.C., Kim J.B., Chin B.R., Kim J.W., Kwon T.G. (2013). Inflammatory granuloma caused by injectable soft tissue filler (Artecoll). J. Korean Assoc. Oral. Maxillofac. Surg..

[52] Lee S.K., Kim S.M., Cho S.H., Lee J.D., Kim H.S. (2015). Adverse reactions to injectable soft tissue fillers: Memorable cases and their clinico-pathological overview. J. Cosmet. Laser Ther..

[53] Lee J.M., Kim Y.J. (2015). Foreign body granulomas after the use of dermal fillers: Pathophysiology, clinical appearance, histologic features, and treatment. Arch. Plast. Surg..

[54] Park T.H., Seo S.W., Kim J.K., Chang C.H. (2012). Clinical outcome in a series of 173 cases of foreign body granuloma: Improved outcomes with a novel surgical technique. J. Plast. Reconstr. Aesthet. Surg..

[55] Lee W., Oh W., Oh S.M., Yang E.J. (2020). Comparative Effectiveness of Different Interventions of Perivascular Hyaluronidase. Plast. Reconstr. Surg..

[56] Kim D.W., Yoon E.S., Ji Y.H., Park S.H., Lee B.I., Dhong E.S. (2011). Vascular complications of hyaluronic acid fillers and the role of hyaluronidase in management. J. Plast. Reconstr. Aesthet. Surg..

[57] Narins R., Jewell M., Rubin M., Cohen J., Strobos J. (2006). Clinical Conference: Management of Rare Events Following Dermal Fillers-Focal Necrosis and Angry Red Bumps. Dermatol. Surg. Off. Publ. Am. Soc. Dermatol. Surg..

[58] Saththianathan M., Johani K., Taylor A., Hu H., Vickery K., Callan P., Deva A.K. (2017). The Role of Bacterial Biofilm in Adverse Soft-Tissue Filler Reactions: A Combined Laboratory and Clinical Study. Plast. Reconstr. Surg..

[59] Sorensen E.P., Urman C. (2015). Cosmetic complications: Rare and serious events following botulinum toxin and soft tissue filler administration. J. Drugs Dermatol..

[60] Zhang L., Zhou Q., Xu H., Gu Q., Shi H., Pan L., Sun Y., Wu S. (2023). Long-term Prognosis of Vision Loss Caused by Facial Hyaluronic Acid Injections and the Potential Approaches to Address This Catastrophic Event. Aesthet. Surg. J..

[61] Sundaram H., Signorini M., Liew S., Trindade de Almeida A.R., Wu Y., Vieira Braz A., Fagien S., Goodman G.J., Monheit G., Raspaldo H. (2016). Global Aesthetics Consensus: Botulinum Toxin Type A--Evidence-Based Review, Emerging Concepts, and Consensus Recommendations for Aesthetic Use, Including Updates on Complications. Plast. Reconstr. Surg..

[62] Edwards P.C., Fantasia J.E. (2007). Review of long-term adverse effects associated with the use of chemically-modified animal and nonanimal source hyaluronic acid dermal fillers. Clin. Interv. Aging.

[63] Lowe N.J., Maxwell C.A., Patnaik R. (2005). Adverse reactions to dermal fillers: Review. Dermatol. Surg..

[64] Beer K., Yohn M., Cohen J.L. (2008). Evaluation of injectable CaHA for the treatment of mid-face volume loss. J. Drugs Dermatol..

[65] Aviv U., Haik J., Weiss N., Berl A., Ofir H., Nardini G., Cleary M., Kornhaber R., Harats M. (2020). Treatment Algorithm for Hyaluronic Acid-Related Complication Based on a Systematic Review of Case Reports, Case Series, and Clinical Experience. Craniomaxillofac. Trauma Reconstr..

[66] Hirsch R.J., Cohen J.L., Carruthers J.D. (2007). Successful management of an unusual presentation of impending necrosis following a hyaluronic acid injection embolus and a proposed algorithm for management with hyaluronidase. Dermatol. Surg..

[67] El-Khalawany M., Fawzy S., Saied A., Al Said M., Amer A., Eassa B. (2015). Dermal filler complications: A clinicopathologic study with a spectrum of histologic reaction patterns. Ann. Diagn. Pathol..

[68] Christensen L., Breiting V., Janssen M., Vuust J., Hogdall E. (2005). Adverse reactions to injectable soft tissue permanent fillers. Aesthetic Plast. Surg..

[69] Shoenfeld Y., Agmon-Levin N. (2011). ‘ASIA’-autoimmune/inflammatory syndrome induced by adjuvants. J. Autoimmun..

[70] Owczarczyk-Saczonek A., Zdanowska N., Wygonowska E., Placek W. (2021). The Immunogenicity of Hyaluronic Fillers and Its Consequences. Clin. Cosmet. Investig. Dermatol..

[71] DeLorenzi C. (2014). Transarterial degradation of hyaluronic acid filler by hyaluronidase. Dermatol. Surg..

